# Low Decision Space Means No Decentralization in Fiji: Comment on "Decentralisation of Health Services in Fiji: A Decision Space Analysis"

**DOI:** 10.15171/ijhpm.2016.82

**Published:** 2016-06-22

**Authors:** Jean-Paul Faguet

**Affiliations:** Department of International Development & STICERD, London School of Economics, London, UK.

**Keywords:** Decentralization, Democracy, Local Government, Good Governance, Civil Society, Fiji

## Abstract

Mohammed, North, and Ashton find that decentralization in Fiji shifted health-sector workloads from tertiary hospitals to peripheral health centres, but with little transfer of administrative authority from the centre. Decision-making in five functional areas analysed remains highly centralized. They surmise that the benefits of decentralization in terms of services and outcomes will be limited. This paper invokes Faguet’s (2012) model of local government responsiveness and accountability to explain why this is so – not only for Fiji, but in any country that decentralizes workloads but not the decision space of local governments. A competitive dynamic between economic and civic actors that interact to generate an open, competitive politics, which in turn produces accountable, responsive government can only occur where real power and resources have been devolved to local governments. Where local decision space is lacking, by contrast, decentralization is bound to fail because it has not really happened in the first place.

## Introduction

This interesting paper examines two periods of the decentralization of healthcare in Fiji, and an intervening recentralization in between. It focuses particularly on the period 2008-2014, comprising the second wave of reform, and asks the question, to what extent were different health functions actually decentralized? The paper employs the ‘Decision Space’ approach pioneered by Bossert,^1^ which defines decision space as the local discretion allowed by central government for functions and sub-functions about financing, service delivery, human resources, and governance exercised by local governments. Carefully operationalized for empirical analysis, measures of decision space allow researchers to investigate the extent to which local authorities have policy discretion, or central authorities effectively circumscribe local choice through rules and incentives that promote central objectives.

Developing these ideas further, Bossert^2^ argues that the decision space approach has several advantages. It puts the focus squarely on the extent to which authority over public choices is shifted from central to local authorities. And it stresses that the choices in question are neither simple not monolithic, but rather involve a range of discretion over different functions and types of decisions. Therefore, we should expect a given decentralization reform to permit more local choice over budgets and financing in some areas (eg, primary education), and hiring and firing in others (eg, public infrastructure). This is a more realistic way of analysing the complexity of real-world experience than the simple decentralized-centralized dichotomies that dominate the literature, concealing more than they reveal. In all these ways, ‘decision space’ is similar to the concept of ‘residual authority’ developed in Faguet.^3^ The empirical operationalizations of decision space, however, constitute an important and original empirical dimension.

The paper finds that although workloads have shifted from tertiary hospitals to peripheral health centres, there has been little transfer of administrative authority from the centre. Decision-making in five functional areas analysed remains highly centralized. Hence – and this in my view is the most important point – the benefits of decentralization for citizens in terms of service provision and real outcomes are likely to be quite limited. The authors rightly stress, however, that the question of real health outcomes is beyond their remit.

Mohammed, North, and Ashton^4^ end their analysis there. The objective of this comment is to push the analysis further. I wish to provide theoretical underpinnings for their final, most powerful conclusion – given more as a speculation – and so show why it is likely true not only for Fiji, but indeed for any country that decentralises in a similar fashion.

Why would decentralization improve government responsiveness to citizen needs in the first place? How, additionally, might it make public services more efficient? The most important thing to understand is that citizen needs, unit costs, and the feasibility of realistic options vary across space. In any field of public policy, different places simply need different things, and have different realistic options available to them. What advantages might local governments have been over central government in such a context?

In comparison to central government, local government is said to benefit from: (*i*) more and/or better information regarding local preferences and conditions; (*ii*) louder citizen voice and participation in the government process; and (*iii*) superior accountability, and, hence, responsiveness of public servants to citizens. All of these effects, it is claimed, come about as a result of the creation of functionally independent local governments that are physically closer to their electorates (than central government), and whose political fortunes are in the hands of those who benefit – or suffer – from the local services they provide. If “bringing government closer to the people” leads to improved information, voice, participation and accountability in public decision-making, then local public services should improve as a result. Services can improve in two broad ways: (*a*) lower costs through greater productive efficiency and less corruption; and (*b*) higher quality, interpreted to include services better-suited to local needs and conditions. Improved services, in turn, should lead to more intensive use by local citizens, and thence to better substantive outcomes. Examples of better substantive outcomes include lower infant mortality rates or higher rates of vaccination.

In practice, will decentralised governments benefit from better information, greater citizen voice, and superior accountability? Or will they be crippled by corruption, elite capture, and low capacity? Literally thousands of studies have attempted to answer this question, with results so mixed that the debate has rumbled on, increasingly fruitlessly, for decades (Faguet and Pöschl 2015; Channa and Faguet Forthcoming).^5,6^ An entire literature has marched into this dead-end, failing to realize that the basic question it posits is misconstrued.^3^ The simplest way to explain it is as follows: The question, “Does decentralization improve service delivery?” has two obvious answers:

(1) **Yes.** Of course services will improve. In at least some localities, resources will be spent and decisions taken in such a way that education, health, and other services improve compared to what central government provided.
(2) **No.** Of course services will worsen. In at least some other localities, resources will be spent and decisions taken in such a way that education, health, and other services worsen compared to what central government did before.

In a third set of localities, which in many countries may be the majority, services will continue much as before, neither significantly better nor worse, and decentralization will have little effect. This is true not by assumption, but by the very definition of decentralization, which even in relatively homogeneous countries should lead to a greater diversity of outcomes, in both type and efficiency. The “outputs” of decentralization are, thus, the simple aggregation of all of the local outputs that reform produces. Each of these local outcomes is, in turn, driven by interactions of the underlying actors and characteristics of each place. The necessary implication is that for much of the past four decades, researchers have been asking the wrong sorts of questions, of a type: ‘Is decentralization good or bad for policy variable X?’ A far better approach is to admit from the outset that decentralization leads to a broad heterogeneity of response, and ask: ‘Why are the good outcomes good, and why are the bad outcomes bad?’ To understand the effects of decentralization on any political and administrative system, we must begin our analysis at the grass roots. To understand decentralization, we must first understand how local government works.

## A Theory of (Local) Government Responsiveness

Why do some local governments provide better services, and others worse ones? Consider Faguet’s model^3,7^ of local governance depicted in the Figure. Our goal is to understand the determinants of responsiveness and accountability in local governance.^ [1]^ As the figure implies, the first-order determinant of a responsive, accountable government is an open, substantively competitive politics. Where politics is open to new entrants and focuses on issues of substance, as opposed to appearance, political competition will produce a strong inclusive tendency in the sense of not leaving significant groups of voters unrepresented. Political platforms will tend to focus on the real needs of voters and firms, rather than descending into beauty contests. Hence, the best predictor of governments that are accountable and responsive is open political systems where competition is substantive and political entrepreneurialism^ [2]^ possible.

**Figure:**
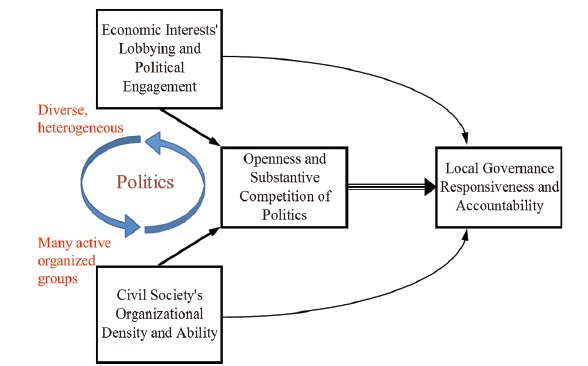
A Theory of (Local) Government Responsiveness. Reproduced from Faguet,^3^ with permission from the University of Michigan Press.

But open, competitive politics is not a fixed municipal characteristic. Rather, it emerges endogenously through the interaction of local economic interests and civic organizations. Where the firms and other economic interests that comprise the local economy are diverse and heterogeneous, and where civil society is organized into many, active groups, and where these two sets of actors interact with one another through local politics, proposing needs, debating competing priorities, and searching for avenues of mutual benefit, local politics will tend strongly towards openness and competition as described above.

Where, by contrast, the local economy is dominated by a single large actor, the diversity of political forms that supports broad representation will tend not to emerge. And where civil society is atomized into individuals, and the intermediating organizations that aggregate preferences and organize collective action are missing, politics will tend to become divorced from society’s needs.^8^ In either case, politics will tend to be less open and less competitive, or competitive in dimensions orthogonal to the needs of voters and the economy. As the figure also implies, economic interests and civic organizations are capable of exerting direct effects on responsiveness and accountability too. But evidence suggests that these effects are weak, and hence firms’ and civic organizations’ primary channel of influence is through the political system.

## Conclusion

The crucial underlying assumption necessary for this system to work is that real power and resources lie in the hands of local authorities. In other words, substantive decentralization has occurred, leaving elected local officials with enough decision space to make the costly competition for power and influence that civic organizations and firms are presumed to engage in worthwhile. If local officials instead have little decision space, implying that power and discretion continue to reside in the centre, then neither firms nor citizen groups will have much incentive to support local parties, lobby officials, or otherwise compete for power. Where public services are concerned, the relevant power will continue to lie in the capital, far beyond the reach of the vast majority of local actors. In such contexts, the requirements of responsive local governments cannot be met, and so the benefits of decentralization cannot be obtained.

This is why the question posed by Mohammed, North, and Ashton is so important. The first step to understanding the effects of any decentralization is to ask Did it really happen? In Fiji, it appears, the answer is no. This is both an important insight for future research, and a clarion call for sincere reform.

## Ethical issues

Not applicable.

## Competing interests

Author declares that he has no competing interests.

## Author’s contribution

JPF is the single author of the paper.

## Endnotes


[1] As opposed to understanding how much power or resources are decentralized, or other systemic aspects of reform that require national-level analysis.

[2] *Political entrepreneurialism* can be defined as the identification, by a new politician or party, of a bloc of voters ill-served by existing political competition. When she then develops proposals and messages attuned to this bloc’s needs, and wins their votes in the following election, she is acting as a political entrepreneur.^3^

